# A new mixture copula model for spatially correlated multiple variables with an environmental application

**DOI:** 10.1038/s41598-022-18007-z

**Published:** 2022-08-16

**Authors:** Mohomed Abraj, You-Gan Wang, M. Helen Thompson

**Affiliations:** 1 School of Mathematical Sciences, Faculty of Science, Queensland University of Technology (QUT), Brisbane, Australia; 2 QUT Centre for Data Science, Brisbane, Australia

**Keywords:** Environmental sciences, Mathematics and computing

## Abstract

In environmental monitoring, multiple spatial variables are often sampled at a geographical location that can depend on each other in complex ways, such as non-linear and non-Gaussian spatial dependence. We propose a new mixture copula model that can capture those complex relationships of spatially correlated multiple variables and predict univariate variables while considering the multivariate spatial relationship. The proposed method is demonstrated using an environmental application and compared with three existing methods. Firstly, improvement in the prediction of individual variables by utilising multivariate spatial copula compares to the existing univariate pair copula method. Secondly, performance in prediction by utilising mixture copula in the multivariate spatial copula framework compares with an existing multivariate spatial copula model that uses a non-linear principal component analysis. Lastly, improvement in the prediction of individual variables by utilising the non-linear non-Gaussian multivariate spatial copula model compares to the linear Gaussian multivariate cokriging model. The results show that the proposed spatial mixture copula model outperforms the existing methods in the cross-validation of actual and predicted values at the sampled locations.

## Introduction

Many environmental sampling is often observed multiple spatially correlated variables at a given geographical location. For instance, multiple topsoil heavy metal concentrations, such as cadmium, zinc, and copper, are sampled from the soil sample at a field location. In forestry, multiple biomass variables, such as bole, foliage, stump, branch, and root biomass, are sampled in a tree. Also, the spatial distribution of forest biomass variables may use to understand wildfire behaviour. These variables can depend on each other in complex ways, such as non-linear and non-Gaussian spatial dependence. The spatial modelling by considering these complex multivariate spatial dependence may increase the prediction accuracy of individual variables, which may help forest managers to minimise risk and save lives. This article focusses on the copula-based spatial modelling of spatially correlated multiple variables and predicts the individual variables while utilising multivariate spatial dependence of spatially correlated variables.

Gaussian-based linear kriging method is widely used to model spatial variables and provides a weighted average measure of linear spatial dependence. The kriging weights do not depend on the different values of samples and also assume linear Gaussian spatial dependence over the spatial domain^1–6^. However, spatial interpolation (prediction or simulation) based on a spatial model expects to behave differently for different values of samples. That is, spatial correlation between samples varies for the different quantiles of samples. Thus, Bárdossy^7^ introduced spatial copula method that can capture the spatial dependence of a spatial variable by considering the different values of samples. In the non-spatial setting, copula method is used to model the dependence between two or more non-spatial variables, which has widely applied in many fields, such as environmental science, finance, economics, medicine and engineering^8–14^. Bárdossy’s^7^ spatial copula method divides the distance over which spatial dependence exists into equally spaced intervals, also referred to as distance classes or spatial bins, and requires the same family of copulas to be fitted across all of the spatial bins. Gräler and Pebesma^15^ proposed a more flexible spatial copula model that permits copulas from different families to be fitted across the distance classes. The added flexibility of Gräler and Pebesma’s model, over Bárdossy’s model, permits increased accuracy in modelling and prediction. The spatial copula concept proposed by Bárdossy, Gräler and Pebesma has used in mining, forestry, soil sampling, hydrology, and other environmental applications^8,16–24^. However, these spatial copula methods enable modelling and predicting a univariate spatial variable without considering the multivariate dependence of spatially correlated multiple variables. Recently, Gnann et al.^25^ improved Bàrdossy’s^7^ method to interpolate a primary spatial variable while considering a secondary correlated spatial variable. However, Gnann et al.^25^ assumed that the joint distribution of primary and secondary variables follows Gaussian copula.

As a solution to model non-Gaussian multivariate spatial dependence in spatial copula framework, Musafer et al.^26^ proposed a multivariate spatial copula model, whereby the correlated spatial variables were transformed into spatially uncorrelated factors using non-linear principal component analysis (NLPCA). Then, Gräler and Pebesma’s univariate spatial copula model was used to model and predict spatially uncorrelated factors. Subsequent back transformation is required to transform predicted values to the scale of the original variables and to re-inject correlation. However, Musafer et al.’s^26^ method indirectly models the joint dependence between spatial variables through a black-box transformation. We directly extend Gräler and Pebesma’s univariate spatial copula to multivariate setting that jointly models spatially correlated multiple variables via a white-box mixture copula^24^. The mixture copula is a joint distribution function of multiple copulas that offers a more flexible framework for parametric statistical modelling and analysis. Also, a single copula family may not be able to capture tail dependencies but the mixture copula capture the tail dependencies as well^24^. The mixture copula has used in the non-spatial setting for modelling multivariate genomic data^27^, and modelling wave height and period^28,29^. We adapt the mixture copula in the spatial setting that offers a more flexible multivariate spatial copula framework for spatially correlated multiple variables.

## Methods

The methodology for modelling spatially correlated multiple variables consists of two essential components: modelling each spatial variable separately using Gräler and Pebesma’s^15^ univariate spatial copula; then joining the univariate spatial copulas using the idea of mixture copula^24^. We also use the proposed spatial mixture model to predict individual variables using inverse conditional approach in a bivariate context^30^. However, the method can be used to predict more than two variables with a trivial generalisation of the bivariate setting.

### Modelling

Let $\mathbf {Z}(x)=[Z_1(x),Z_2(x),\ldots ,Z_m]$ be the second-order stationary multivariate spatial random field $\mathbf {Z}$ with *m* spatial variables that are sampled at the same two-dimensional location $x\in \mathcal {X}$, and let $X=(x_1,x_2,\ldots ,x_n)$ be the set of existing locations in the given spatial domain $\mathcal {X}$.

A spatial copula^15^ describes the joint spatial dependence of a univariate spatial variable at any two spatial locations *x* and $x+h$, where *h* is the separation distance between two locations. Hence, spatial copulas model dependence of one spatial location relative to another spatial location, rather than modelling dependence using absolute locations.

**Figure 1:**
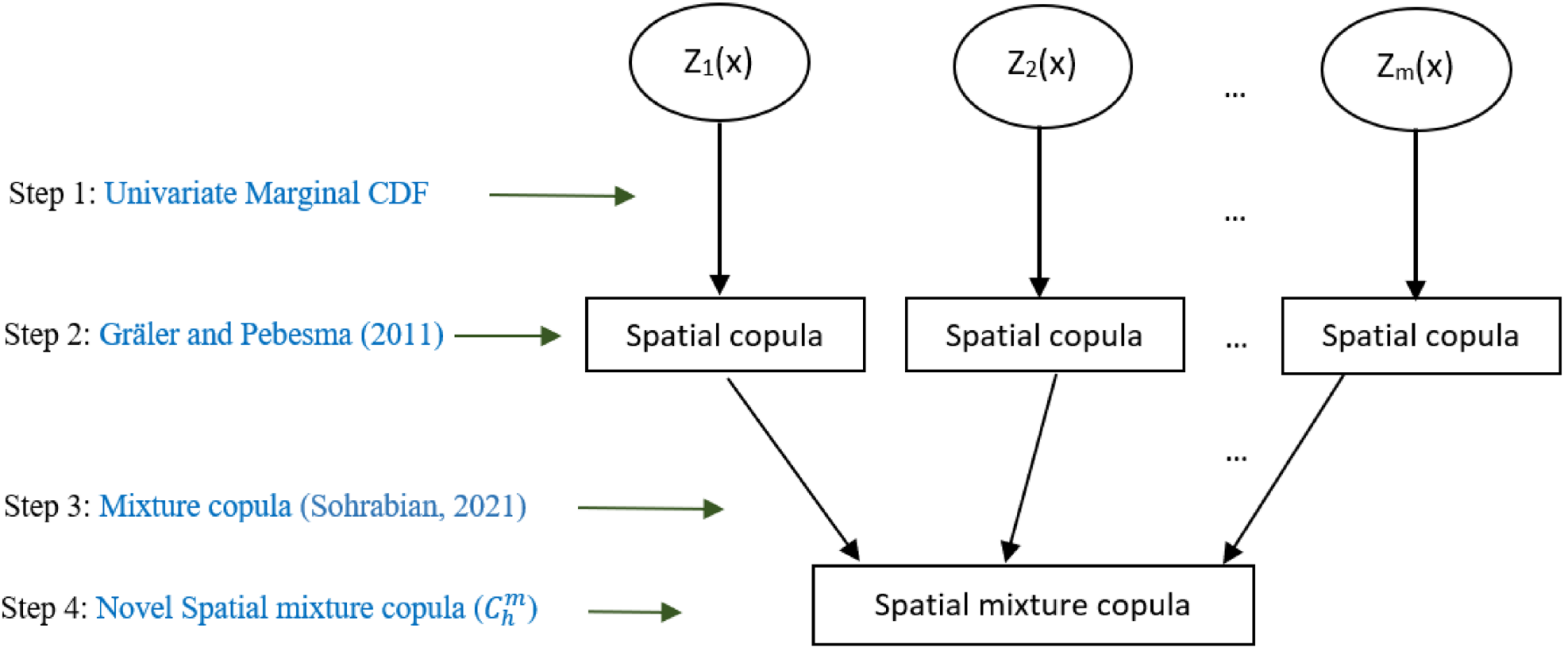
A diagram for spatial mixture copula construction.

The methodology for modelling spatially correlated multiple variables is simply shown in Fig. 1, and a detail procedure for the model development is provided in steps 1–4.

**Step 1:** For each spatial variable $Z_l, l=1,2,\ldots ,m$, models the marginal cumulative distribution functions (CDFs), such as Gamma, Weibull, Normal, Log-normal, and obtain the best fitted CDFs. Let $F_l$ denote the best fit CDF of $Z_l$, which is assumed to be same at each location *x*, i.e., $F_l(Z_l(x))=F_l(Z_l(x+h))$.

The proposed method is based on the concept of distance dependent spatial copula^15,31^. Hence, the distances between every pair of locations are calculated. Suppose, $x_1$, $x_2$, $x_3$ and $x_4$ are four sampled locations of ***Z***.

**Figure 2:**
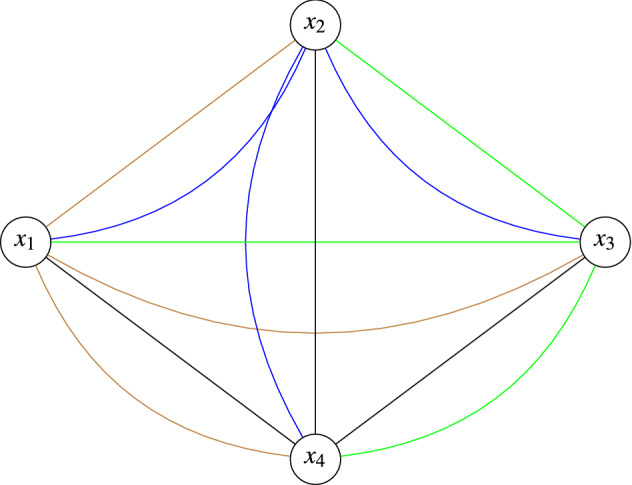
Example plot to show the possible pairs with four locations.

As given in Fig. 2, the distances are calculated for each location pair $\{x_i$-$x_j=h\}$ as $\sqrt{(a_i-a_j)^2 + (b_i-b_j)^2}$, where $(a_i,b_i)$ and $(a_j,b_j)$ are the coordinates of $x_i$ and $x_j$, for $i\ne j$, $\forall i,j = 1,2,\ldots ,n$, respectively. Also, $n(n+1)/2$ number of pairs are obtained with *n* sampled locations. The next important step of the methodology is spatial binning.

The spatial dependence of copula-based spatial models depends on the distance between locations. As the distance between two points increases, spatial dependence between points decreases until it is independent or negligible enough to be considered independent. The distance at which independence occurs is referred to as the cut-off distance and is determined empirically using a correlogram. The correlogram plots the Kendall’s tau $\tau$ correlation coefficient for each spatial bin, and a curve is fitted through the plotted points. Similar to a variogram in Kriging, the cut-off distance is visually determined as the distance at which the curve plateaus.

**Figure 3:**
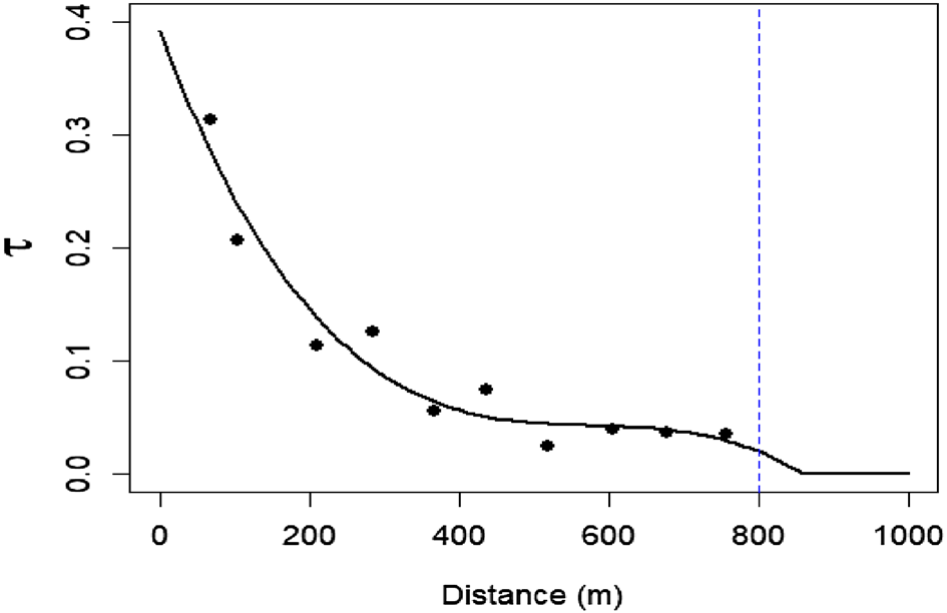
An example correlogram. The blue dashed line indicates the upper limit of cut-off distance at which pairs of points are no longer considered to be spatially dependent. Empirical $\tau$ values (black dots) overlaid with theoretical cubic smooth line.

**Step 2:** Based on the distance between pairs, place each sample pair $\{F_l(Z_l(x),F_l(Z_l(x+h))\}$ into *K* equally spaced spatial bins as follows: $[0,h_1),[h_1,h_2),\ldots ,[h_{K-1},h_K)$, where $h_K$ is the cut-off distance. A correlogram is used to determine the cut-off distance as a plot of $\tau$ against the mean distance of each bin, which is calculated using the pairs belonging to relevant spatial bin. Figure 3 depicts an example correlogram.

Given the pairs of points for each spatial bin, spatial copula that describes the dependence of spatial variable $Z_l$ at any two locations can be calculated as,

1 $$\begin{aligned} \begin{aligned} C_{l,k,h}(u,v)&= P[F_l(Z_l(x)) \le u,F_l(Z_l(x+h)) \le v ],\\&= C_{k}(F_l(Z_l(x)),F_l(Z_l(x+h))), \end{aligned} \end{aligned}$$

where $k=1,2,\ldots ,K$ is the index of the spatial bin, *u* and *v* are any selected quantiles of the corresponding univariate CDF of $Z_l$ at locations *x* and $x+h$.

The copulas for each bin are selected using maximum log-likelihood values of competing copulas, such as Gaussian, Student’s t, Clayton, Frank, Gumbel, and Joe^30^, which represent variety of dependence structures. Then, a mixture copula is used to determine the multivariate spatial dependence across bins as a weighted linear combination of copula.

**Step 3:** For each spatial bin *k*, use the spatial copulas in Eq. (1) to construct the mixture copula $C^m_{k,h}$ as,

2 $$\begin{aligned} C^m_{k,h}(u,v) = \sum _{l=1}^{m} w_lC_{l,k,h}, \end{aligned}$$

where, $w_l$ is the mixture weight, $\sum _{l=1}^{m} w_l=1$, and $0< w_l <1$.

An equal weight can be used in Eq. (2) if the correlogram of each variable is not significantly different. Otherwise, compare different weight combinations across bins and obtain the optimal weight combination. Moreover, for small distances, pairs of points will become extremely strong dependent and modelled using a comonotonic copula *M*(*u*, *v*). For large distances, pairs will become independent and modelled using a product copula $\Pi (u,v)$^32^, as follows

$$\begin{aligned} M(u,v) := min \{u,v\} \quad when \quad h \rightarrow 0, \quad \Pi (u,v) := uv \quad when \quad h \rightarrow \infty . \end{aligned}$$

The mixture copula in Eq. (2) only describes the multivariate spatial dependence across individual bins. However, a spatial model should be able to capture spatial autocorrelation between bins^15^. For instance, points near the upper bound of the first bin and the lower bound of the second bin may have similar features; points near the upper bound of the second bin and lower bound of the third bin; and so on. Thus, the spatial dependence is incorporated using the distance dependent parameter $\lambda _k$ that determines spatial dependence while incorporating spatial autocorrelation.

In practical situations, the first bin is modelled using the best fit copula for that bin, and subsequent bins are modelled using the convex linear combination of copulas with parameter $\lambda _k$^15^. Further, pairs that fall above the cut-off distance are often omitted and not incorporated into the convex combination, assumed as an independent copula.

**Step 4:** Use Eq. (2), construct the distance dependent spatial mixture copula of $\mathbf {Z}$ as the convex linear combination of mixture copulas of each spatial bin as follows,

3 $$\begin{aligned} C^m_{h}(u,v)={\left\{ \begin{array}{ll} C^{m}_{1,h}(u,v), &{} {0 \le h<h_1},\\ (1-\lambda _{2})C^{m}_{1,h}(u,v) + \lambda _{2} C^{m}_{2,h}(u,v), &{} {h_1 \le h<h_2},\\ \vdots &{}\vdots \\ (1-\lambda _{K})C^{m}_{K-1,h}(u,v) + \lambda _{K} C^{m}_{K,h}(u,v), &{} {h_{K-1} \le h < h_K},\\ uv ,&{} {h_K \le h }, \end{array}\right. } \end{aligned}$$

where $\lambda _{k} = \frac{\bar{h}_k-h_{k-1}}{h_k - h_{k-1}}$ for $k=2,3,\ldots ,K$, $\bar{h}_k$ is the mean distance, and $h_1,h_2,\ldots ,h_K$ denote upper limits of the chosen distances for the spatial bins.

### Prediction

Prediction of individuals spatial variables at sampled locations based on the spatial mixture copula is described in a bivariate context. That is $m=2$, then $C^m_h$ is the spatial mixture copula of $Z_1$ and $Z_2$. The prediction method demonstrates the advantage of using a secondary correlated spatial variable in the prediction of a primary spatial variable^25^. Thus, an inverse conditional prediction approach is proposed^30^,[pp. 40–42] where a secondary correlated spatial variable is known when predicting the primary spatial variable .

Suppose $Z_1$ is the primary variable of interest, then $Z_2$ is correlated secondary variable. The prediction of $Z_1$ at location *x* conditional on the known given value of $Z_2$ can be generated at the same location *x*, using the copula CDF of $C^m_h$. The procedure of the inverse conditional approach is given in steps 5-8,

**Step 5:** Obtain the joint CDF values of $Z_1$ and $Z_2$ using $C^m_{h}$, and let *T* be the vector with joint CDF values.

**Step 6:** Obtain the marginal CDF values of $Z_2$ using $F_2$, and let *R* be the vector with marginal CDF values.

**Step 7:** Derive the conditional distribution of *T*, given $R=r$, using the partial derivative of $C^m_h$ as follows,

$$\begin{aligned} \begin{aligned} C^m_{h,r}(T|R=r)&= P[T \le t|R=r],\\&=\frac{\partial }{\partial r}C^m_h(t,r), \end{aligned} \end{aligned}$$

let $s = (C^m_{h,r})^{-1}(T|R=r)$ be the conditional predicted value of $Z_1$ at location *x*.

**Step 8:** Take, $$Z_1 = F_1^{-1}(s)$$.

The prediction of $Z_2$, given $Z_1$, can be described by simply switching the subscripts 1 and 2 in the steps 5–8. The proposed method can be validated against actual values at sampled locations by cross-validation, and three scenarios are considered with the existing methods.

- Can any improvement in the prediction of individual variables be gained by utilising the multivariate spatial dependence using mixture copula over the univariate pair copula^15^?
- Can any improvement in the prediction of individual variables be gained by utilising the mixture copula over the NLPCA transformation based spatial copula^26^?
- Can any improvement in the prediction of individual variables be gained by utilising the non-linear non-Gaussian multivariate spatial dependence (spatial mixture copula) over the linear Gaussian multivariate spatial dependence (cokriging)^33^?

The cross-validation study is illustrated using mean absolute error (MAE), root mean square error (RMSE), mean absolute percentage error (MAPE). The MAE, RMSE and MAPE can be calculated using the actual and predicted values at the sampled locations^26^. Also, accuracy in the reproduction of the bivariate relationship of $Z_1$ and $Z_2$ is evaluated based on the mean square error from the kernel density estimation (KDE MSE). The KDE MSE can be calculated by taking the mean of the squared differences between the bivariate KDEs of the actual and predicted data^26^.

## Application

The proposed method was applied to model real forest data that was taken from georeferenced forest inventory plots in the US Department of Agriculture Forest Service Bartlett Experimental Forest (BEF) in Bartlett, New Hampshire^34^. The variables of interest were forest-wide biomass estimations within the area of 1053 hectares (measured in mg/ha). In this study, only foliage biomass ($Z_1$) and bole biomass ($Z_2$) were used that sampled at 335 two-dimensional locations.

The prediction of bole biomass can be used for carbon accounting purposes, and the prediction of foliage biomass can be used to identify regions with high values of foliage biomass. Also, the behaviour of wildfires depends on pools of biomass variables^26,35^.

**Figure 4:**
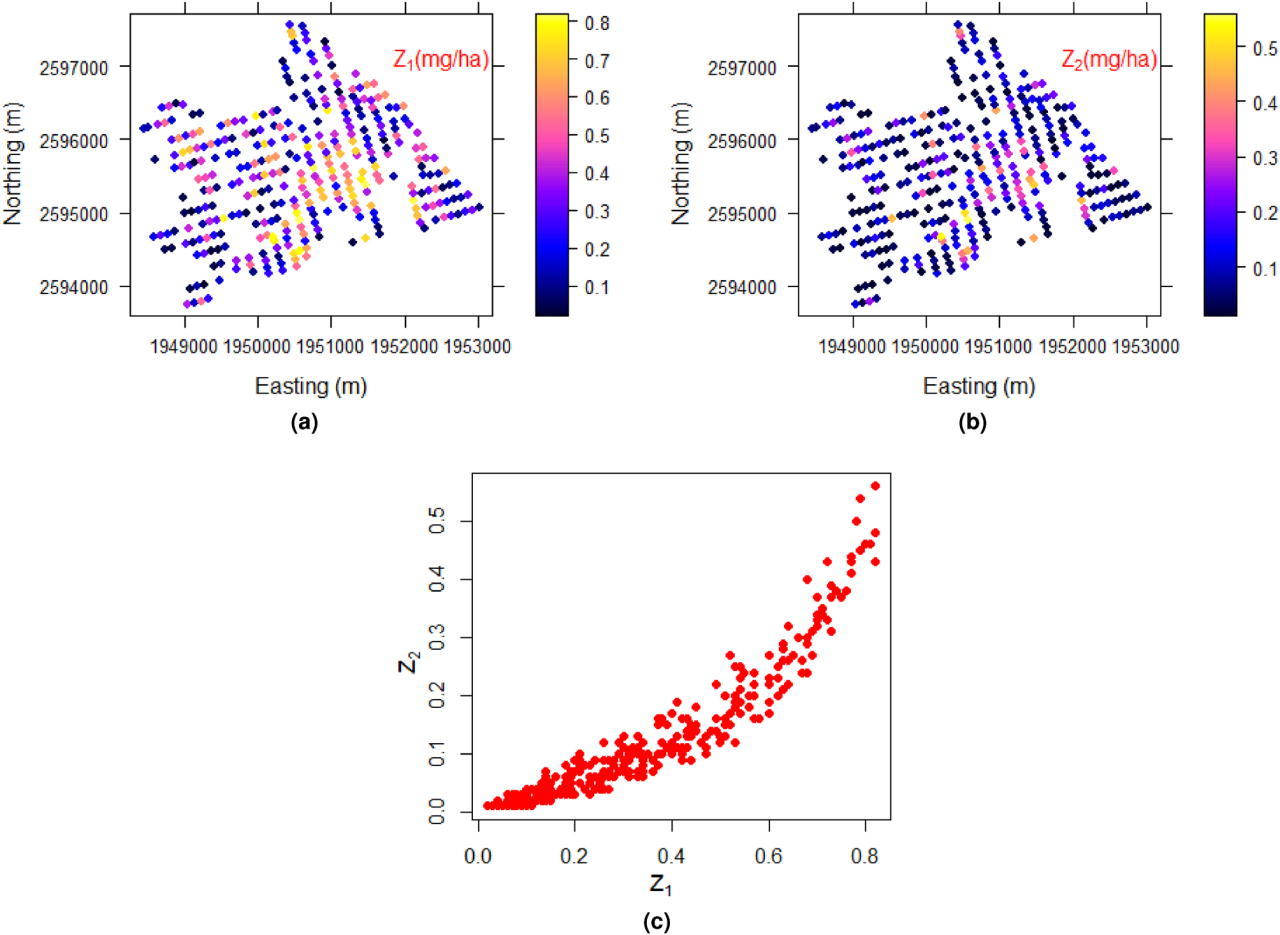
BEF data. Spatial distributions of (**a**) $Z_1$, (**b**) $Z_2$, and (**c**) scatter plot between $Z_1$ and $Z_2$.

Table 1 gives the summary statistics of the data. Figure 4a,b show the spatial distributions of $Z_1$ and $Z_2$ at observed locations. Figure 4c shows a strong bivariate non-linear relationship between $Z_1$ and $Z_2$. The best marginal distributions were selected based on the maximum log-likelihood (ML) values. The Weibull distribution was achieved as the best distribution for $Z_1$ based on the ML values, 65.03, **69.43**, 33.26, 44.03, and the Gamma distribution was achieved as the best distribution for $Z_2$ based on the ML values, **378.86**, 378.28, 250.20, 377.51, of Gamma, Weibull, Normal, Log-normal distributions respectively. Then, the CDF values of $Z_1$ and $Z_2$ were calculated using the corresponding CDFs. The following steps for the modelling is only incorporated the CDF values of $Z_1$ and $Z_2$ (Step 1).

**Table 1:** Summary statistics of $Z_1$ and $Z_2$.

Statistics	$Z_1$	$Z_2$
*n*	335	335
Mean	0.334	0.119
Standard deviation	0.219	0.115
Minimum	0.200	0.010
First quartile $Q_1$	0.140	0.030
Median	0.300	0.090
Third quartile $Q_3$	0.510	0.160
Maximum	0.820	0.560

**Table 2:** BEF data: spatial binning.

Bins	Mean distance	Kendall’s tau	
		$Z_1$	$Z_2$
0–80	68	0.31	0.23
80–160	105	0.20	0.18
160–240	209	0.11	0.10
$\vdots$	$\vdots$	$\vdots$	$\vdots$
720–800	758	0.03	0.03

The cut-off distance was selected as 800 m using the correlograms of variables, and ten equally spaced (80 m) spatial bins were created (see Table 2). Table 3 shows the best fit copulas and the estimated copula parameters, where $C_{1,k,h}$ and $C_{2,k,h}$ are the fitted univariate spatial copulas of $Z_1$ and $Z_2$ respectively (Step 2). The correlation across bins almost similar for each variable (see Table 2), and then equal weights were used. Table 4 shows the mixture copulas of each bin (Step 3).

**Table 3:** The univariate spatial copulas for each bin.

Bins	${C_{1,k,h}}-Z_1$	${C_{2,k,h}}-Z_2$
0–80	$C_{1,1,h}$ = Joe (1.71)	$C_{2,1,h}$ = Joe (1.48)
80–160	$C_{1,2,h}$ = Gumbel (1.31)	$C_{2,2,h}$= Gaussian (0.29)
160–240	$C_{1,3,h}$ = Gumbel(1.16)	$C_{2,3,h}$ = Frank (1.12)
240–320	$C_{1,4,h}$ = Gumbel (1.10)	$C_{2,4,h}$ = Clayton (0.19)
320–400	$C_{1,5,h}$ = Gumbel (1.06)	$C_{2,5,h}$= Clayton (0.13)
400–480	$C_{1,6,h}$ = Joe(1.09)	$C_{2,6,h}$ = Joe (1.08)
480–560	$C_{1,7,h}$ = Joe (1.07)	$C_{2,7,h}$ = Gumbel (1.03)
560–640	$C_{1,8,h}$ = Clayton (0.09)	$C_{2,8,h}$= Clayton (0.06)
640–720	$C_{1,9,h}$ = Clayton(0.08)	$C_{2,9,h}$ = Clayton (0.05)
720–800	$C_{1,10,h}$ = Joe (1.05)	$C_{2,10,h}$ = Gumbel (1.03)

**Table 4:** The mixture copulas of each bin with $w_1$=$w_2$=0.5.

Bins	Mean distance ($\bar{h}_k$)	Mixture copula ($C^{m}_{k,h}$)
0–80	68	$C^{m}_{1,h} = 0.5C_{1,1,h} +0.5C_{2,1,h}$
80–160	105	$C^{m}_{2,h}=0.5C_{1,2,h} + 0.5C_{2,2,h}$
160–240	209	$C^{m}_{3,h} = 0.5C_{1,3,h} + 0.5C_{2,3,h}$
240–320	284	$C^{m}_{4,h} = 0.5C_{1,4,h} + 0.5C_{2,4,h}$
320–400	368	$C^{m}_{5,h} = 0.5C_{1,5,h} +0.5C_{2,5,h}$
400–480	436	$C^{m}_{6,h} =0.5C_{1,6,h} + 0.5C_{2,6,h}$
480–560	518	$C^{m}_{7,h} = 0.5C_{1,7,h} + 0.5C_{2,7,h}$
560–640	606	$C^{m}_{8,h} = 0.5C_{1,8,h} + 0.5C_{2,8,h}$
640–720	678	$C^{m}_{9,h} = 0.5C_{1,9,h} +0.5C_{2,9,h}$
720–800	758	$C^{m}_{10,h} = 0.5C_{1,10,h} + 0.5C_{2,10,h}$

The mixture copulas in Table 4 were used to develop the distance dependent convex combination of mixture copulas as given in the Eq. (3), which is the proposed spatial mixture copula of the spatially correlated $Z_1$ and $Z_2$ (Step 4), is given by

$$\begin{aligned} C^m_{h}(u,v)={\left\{ \begin{array}{ll} C^{m}_{1,h}, &{} {0 \le h< 80 },\\ 0.69C^{m}_{1,h} + 0.31C^{m}_{2,h}, &{} {80 \le h< 160 },\\ 0.39C^{m}_{2,h} + 0.61C^{m}_{3,h}, &{} {160 \le h< 240 },\\ 0.45C^{m}_{3,h} + 0.55C^{m}_{4,h}, &{} {240 \le h< 320 },\\ 0.40C^{m}_{4,h} + 0.60C^{m}_{5,h}, &{} {320 \le h< 400 },\\ 0.55C^{m}_{5,h} + 0.45C^{m}_{6,h}, &{} {400 \le h< 480 },\\ 0.51C^{m}_{6,h} + 0.49C^{m}_{7,h}, &{} {480 \le h< 560 },\\ 0.42C^{m}_{7,h} + 0.58C^{m}_{8,h}, &{} {560 \le h< 640 },\\ 0.52C^{m}_{8,h} + 0.48C^{m}_{9,h}, &{} {640 \le h< 720 },\\ 0.52C^{m}_{9,h} + 0.48C^{m}_{10,h}, &{} {720 \le h < 800 },\\ uv,&{} {800 \le h }, \end{array}\right. } \end{aligned}$$

where $$\lambda _2$$ = $$\frac{105-80}{160-80}$$ = 0.31, $$\lambda _{3}$$ = 0.61,$$\ldots$$, $$\lambda _{10}$$ = 0.48.

**Figure 5:**
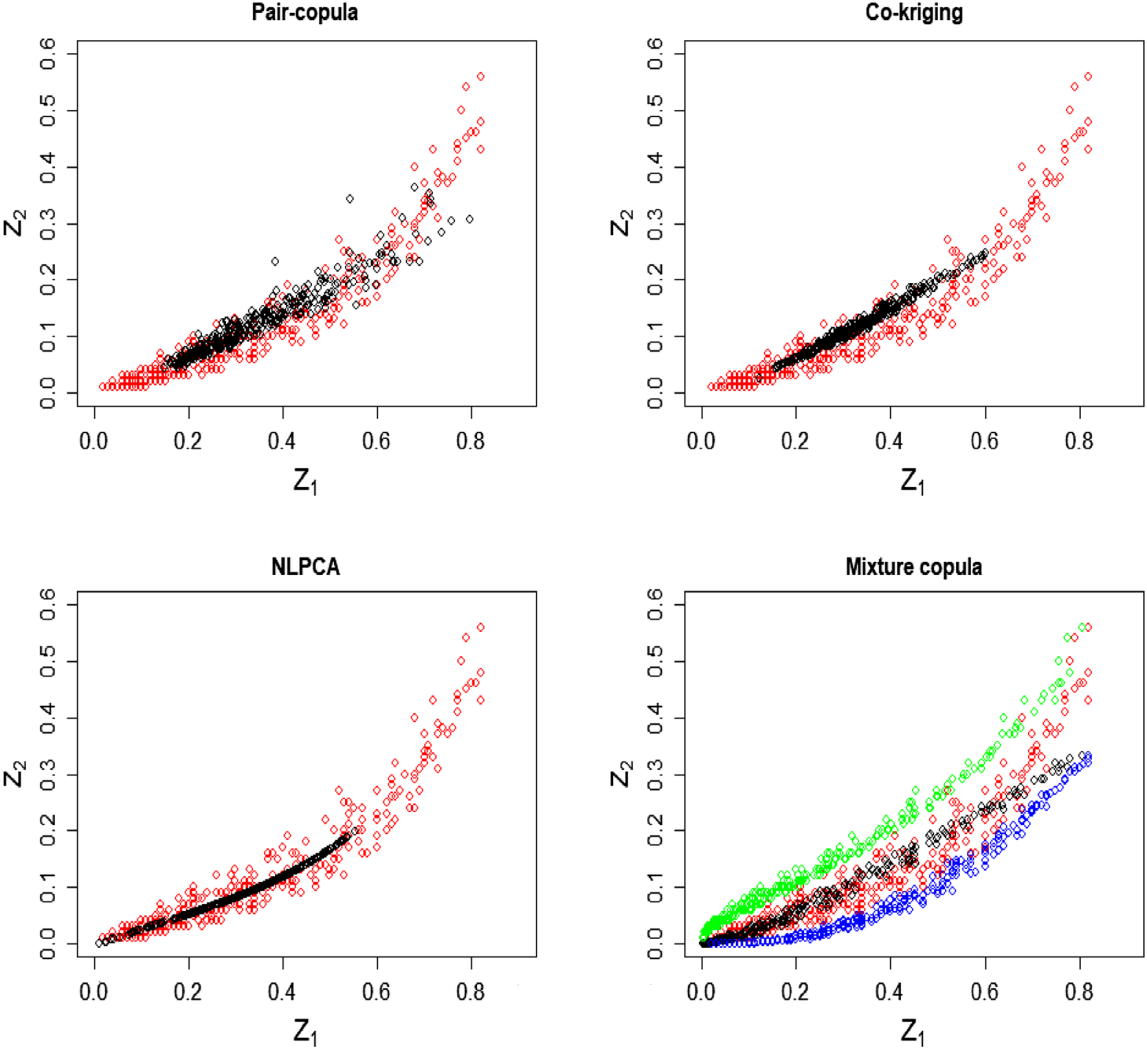
Reproduction of bivariate relationship using various methods. Actual (red), predicted (black), $Z_1$ given $Z_2$ (green), and $Z_2$ given $Z_1$ (blue).

The proposed spatial mixture copula method was used to predict $Z_1$ and $Z_2$ using the inverse conditional approach as described in the steps 5–8. Figure 5 shows the bivariate relationship of $Z_1$ and $Z_2$. Table 5 shows the model validation results with the existing methods.

**Table 5:** Model validation in prediction of $Z_1$ and $Z_2$.

Method	$Z_1$			$Z_2$			KDE
	RMSE	MAE	MAPE	RMSE	MAE	MAPE	MSE
Pair copula	0.20	0.17	1.12	0.11	0.08	1.98	3.61
Cokriging	0.19	0.16	**0.54**	0.11	0.08	0.75	3.71
NLPCA	0.29	0.24	1.64	0.14	0.10	2.21	12.40
Mixture copula	**0.14**	**0.13**	**0.56**	**0.06**	**0.05**	**0.63**	**1.34**

Significant values are in [bold].

According to Table 5 almost all the RMSE, MAE, and MAPE values are the lowest for the $Z_1$ and $Z_2$ predictions based on the spatial mixture copula. The MAPE value of cokriging method is the smallest for the $Z_1$ prediction that is very close to the spatial mixture copula. Thus, it can be seen that the proposed method outperformed in the prediction of $Z_1$ and $Z_2$ across the observed locations. Also, the proposed method accurately reproduces the bivariate relationship in terms of the minimum value of KDE MSE.

In Fig. 5, the univariate pair copula method does not reproduce the tail values of both variables. Cokriging is unable to predict tail values and follows a strictly linear relationship. Although the NLPCA spatial copula method reproduces the non-linear relationship, it cannot reproduce upper tails, specifically for $Z_2$. The prediction of individual variables using the novel spatial mixture copula method accurately predicts both upper and lower tail values, and conditional values of the variables reproduce the non-linear relationships between them. Thus, using mixture copula in the multivariate spatial copula framework is improved the accuracy in the univariate prediction.

## Conclusions

This article proposed a new mixture copula method for modelling spatially correlated multiple variables. The proposed method models multiple spatial variables without any normalisation of the original variables, such as NLPCA transformation. The method was applied to model bivariate non-linear spatial variables $Z_1$ (foliage biomass) and $Z_2$ (bole biomass). The model performance was assessed in the cross-validation of actual versus predicted values at sampled locations. The use of multivariate spatial dependence in the univariate prediction, the strength of the mixture copula in the univariate prediction, and utilising non-linear non-Gaussian multivariate spatial dependence in the univariate prediction, were compared with the existing univariate pair copula, NLPCA spatial copula and cokriging methods, respectively. The results showed that the proposed spatial mixture copula model outperformed the existing methods in terms of the minimum values of RMSE, MAE, MAPE, and KDE MSE.

The method also applied to non-linear simulated bivariate correlated variables (see  Supplementary online), where the spatial mixture copula outperformed the existing methods, in terms of predicting individual simulated variables and their bivariate relationship. The proposed method used equal weights for each variable for both BEF application and simulation study. However, further improvement to the spatial mixture model is the optimal weights selection of each variable in the mixture copula modelling. For instance, one spatial variable may have a strong spatial dependence across locations than the other variable, and the optimal weights selection may increase the prediction accuracy of each variable across locations. The prediction method is explained for the bivariate case (m = 2), however, it can be extended to multivariate setting. Also, the proposed spatial mixture copula can be extended to multivariate spatial sampling design methodology for optimally selecting additional sampling to reduce prediction uncertainty by leveraging spatially correlated multiple variables. Moreover, the proposed method assumes isotropic spatial dependence of spatial variables but can be extended to model spatially correlated anisotropic variables, which can be present in mining^36^ and soil variables^37^, for example.

The proposed method assumes that the spatial random field is stationary. However, the method can be extended to non-stationary spatial processes. For example, a non-stationary spatial process can be divided into several locally stationary processes. A univariate spatial copula can be modelled to each stationary process, and then a global non-stationary spatial copula can be constructed as a mixture of locally stationary spatial copulas. Furthermore, the proposed method assumes that all data points are known and collected at the same set of locations. However, measurements could be unavailable or difficult to sample at some locations, which is quite common in functional spatial data analysis. Thus, the proposed method can be extended for modelling and predicting complex functional spatial data.

## Supplementary Information

Supplementary Information.

## Data Availability

In implementing the proposed model in this paper, R software version 3.6.3 (R Development Core Team 2020) was used. Specifically, R package “spcopula” version 0.2-4 of Gräler was entirely used in this study (see http://r-forge.r-project.org/projects/spcopula/), including key dependent packages, such as “copula”, “VineCopula” version 2.1.8, “sp”, “spBayes” “MASS”,“fitdistrplus”. The data is available in “spBayes” package, where only non-zero values of biomass were considered in this study. For simulation study, “gstat” package was mainly used that facilitated the unconditional prediction of Gaussian random fields. Moreover, in comparing the proposed model with the most relevant NLPCA spatial copula model, MATLAB software was used, specifically “Nonlinear PCA toolbox” of Scholz (see http://www.nlpca.org/matlab.html)^38^. Data for developed method can be found in Dropbox: https://www.dropbox.com/s/cdbmx89fgjul9cg/bef_data.csv?dl=0.
