# Supplementary material for: A new mixture copula model for spatially correlated multiple variables with an environmental application
Source: Sci Rep. 2022 Aug 16;12:13867. doi: 10.1038/s41598-022-18007-z (PMC9381801; doi:10.1038/s41598-022-18007-z)
Supplement: Supplementary file 1 — Supplementary Information. [file 41598_2022_18007_MOESM1_ESM.pdf]

Simulation study

A simulation study was proposed to assess the performance of the novel spatial mixture copula method with an artificial non-linear bivariate correlated variables. The simulation of spatial variables using copula method is complex, because simulated data should satisfy the fundamental property of geostatistics. That is, the dependence between pairs of points is strong for nearby points and it decreases when the distance between points increases. Hence, an existing ordinary kriging simulation method was modified to simulate non-Gaussian and non-linear bivariate correlated variables. The summary of the simulation study is given in steps a–c.

a) Create a 100 by 100 two-dimensional grid of data locations, denote coordinates belong to domain Ω

b) Create an exponential variogram model with artificial parameters as given in Fig. S1,

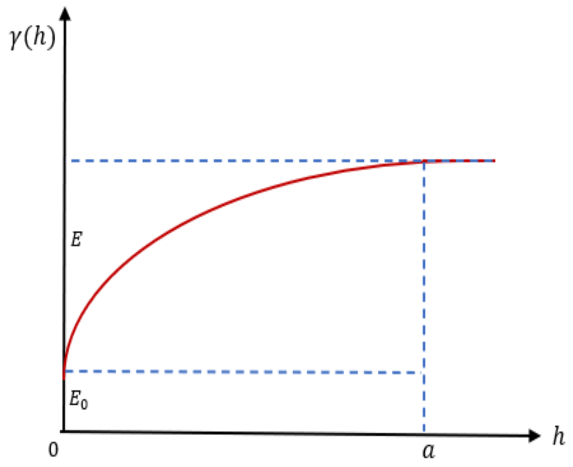

Figure S1: Exponential variogram with nugget (E_0), partial sill (E), and range (a).

The semi-variance $\gamma(h)$ with distance h is calculated as

$$\gamma(h) = E_0 + E\Big[1 - exp\Big(-\frac{h}{a}\Big)\Big],$$

and ordinary kriging can be used to predict an unobserved value at an unsampled location x_0 as follows

$$\hat{Z}(x_0) = \sum_{i=1}^{n} \beta_i Z(x_i),$$

where $Z(x)$ is a univariate isotropic spatial random field and β_i are weights that determined from the exponential variogram.

Here, we used the ordinary kriging method to simulate 10000 univariate Gaussian variables over the 100 by 100 girded locations. Then, randomly select 100 samples from each Gaussian variables, where $\omega \in \Omega$ is corresponding random locations. The procedure of simulating non-linear and non-Gaussian spatially correlated variables is shown in Algorithm 1.

c) Fit the proposed spatial mixture copula C_h^m for each simulated bivariate non-linear non-Gaussian correlated variables, and simultaneously predict each variable at location ω using the inverse conditional method explained in the steps 5–8.

Algorithm 1 Algorithm for simulating spatially correlated artificial variables

Definition and Notation:

Let $\mathbf{S}$ be the matrix with 10000 simulated Gaussian variables with sample size 100

i is the index of first variable

j is the index of second variable

$S_{1,i} = NULL$ # vector to store the first non-Gaussian variables

$S_{2,j} = NULL$ # vector to store the second non-Gaussian variables

$S_{1,i}(\omega)$ is the ith simulated variable at location $\omega \in \Omega$

$S_{2,j}(\omega)$ is the jth simulated variable at location $\omega \in \Omega$

$S_{1,i}(\omega)$ and $S_{2,j}(\omega)$ are vectors with sample size 100, i.e., $\omega = (\omega_1, \omega_2, \ldots, \omega_{100})$

Calculation:

while (100 non-Gaussian bivariate spatial variables are simulated){

for i in 1 to 10000

while ($S_{1,i}$ *with desired spatial correlation structure is obtained - see step 2 in the method)*

$S_{1,i} = \mathbf{S}[, i]$ + noise # e.g., normal distribution with mean = 2 and sd = 0.01

Calculate the best marginal distribution of $S_{1,i}$ (a non-Gaussian marginal distribution)

Create the spatial bins of $S_{1,i}$ (see step 2)

end while

end for

for j in 1 to 10000

while ($S_{2,j}$ *with desired spatial correlation structure is obtained)*

$S_{2,j} = (S_{1,i})^p$ + noise # a polynomial degree p to obtain a strong non-linear relationship

Calculate the best marginal distribution of $S_{2,j}$ (a non-Gaussian marginal distribution)

Create the spatial bins for $S_{2,j}$

end while

end for

} *end while*

Table S1 gives the cross-validation results of the simulation study, which compares the mean RMSE, and standard error (SE) in the prediction based on 100 simulated samples with the existing methods. Fig. S2 shows a graphical summary of the bivariate relationship of the simulation study.

**Method**	**RMSE**			
	**Mean (S_1)**	**SE**	**Mean (S_2)**	**SE**
Pair-copula	0.809	0.010	78.370	2.020
Co-kriging	0.780	0.010	78.360	1.960
NLPCA	1.130	0.020	104.905	3.220
Mixture copula	**0.630**	0.010	**47.980**	1.220

Table S1: Model validation of artificial non-linear bivariate correlated variables

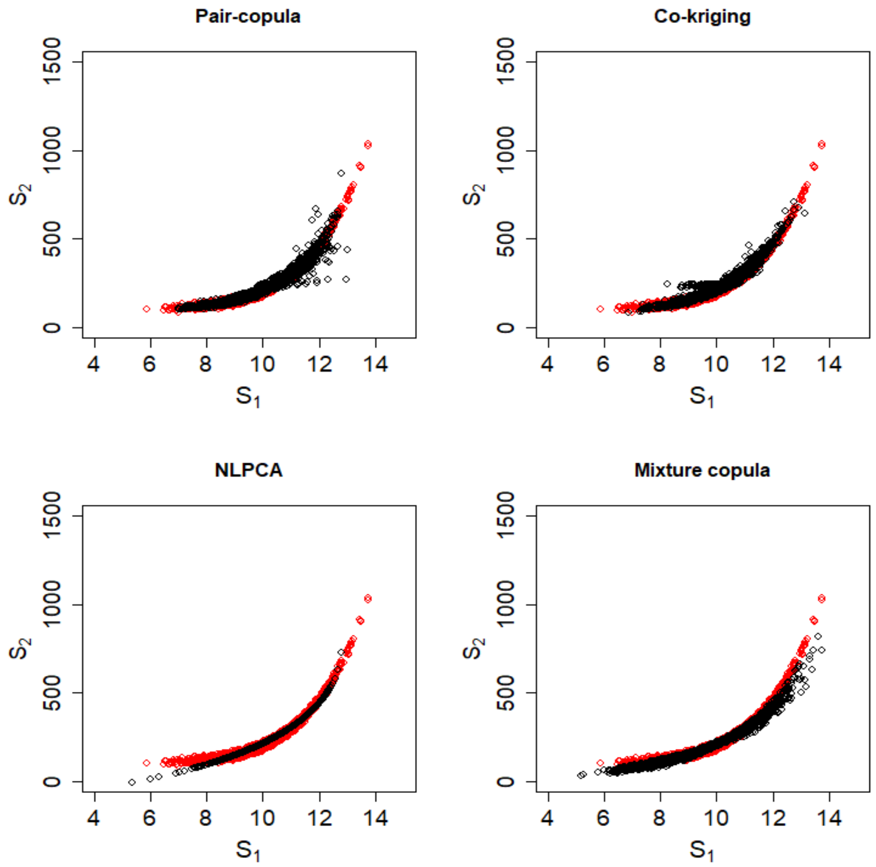

Figure S2: Relationship between first simulated variable (S_1) and second simulated variable (S_2), 100 actual simulated values (red) overlay with 100 predicted simulated values (black).

According to Fig. S2, the cokriging reproduces the non-linear relationship because the simulation study is based on the kriging method. The pair copula and the NLPCA methods fail to predict tails of the actual data. Also, the NLPCA method always predicts very similar values for different simulated variables because Artificial Neural Network used in NLPCA transformation produces very similar uncorrelated factors. However, the spatial mixture copula method more accurately reproduces the non-linear relationship when compared to the other methods because the mixture copula completely utilises the actual relationship between variables (see also conditional prediction plots in Figs. S3, S4). Thus, the model validation results in Table S1 based on the spatial mixture copula achieves the minimum mean RMSE.

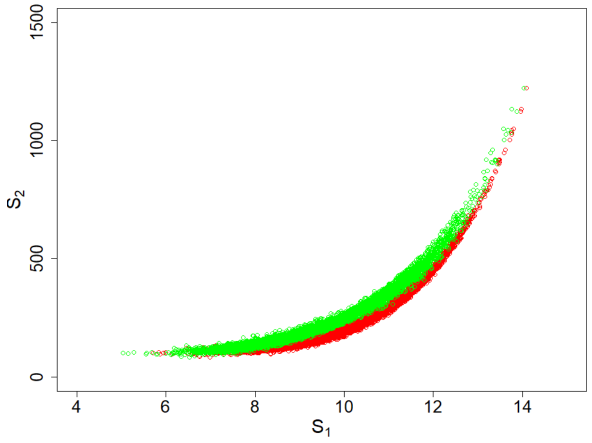

Figure S3: Conditional prediction: Actual (red); S_1 given S_2 (green).

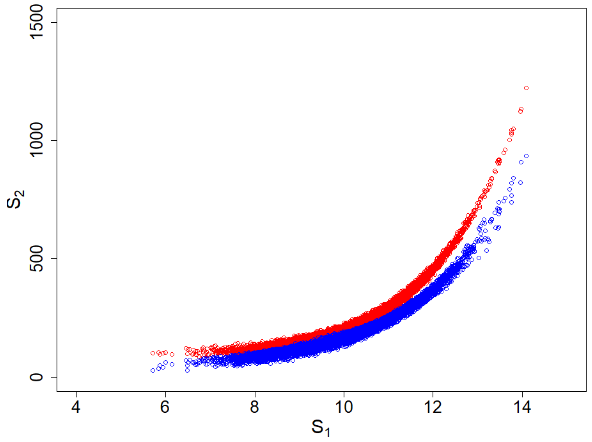

Figure S4: Conditional prediction: Actual (red); S_2 given S_1 (blue).
